# Treatment of Recurrent Total Hip Arthroplasty Dislocation Caused by Distorted Proximal Femoral Anatomy Due to a Previously Healed Trochanteric Fracture

**DOI:** 10.7759/cureus.29969

**Published:** 2022-10-06

**Authors:** Christos I Vosinakis, Ioannis C Vossinakis

**Affiliations:** 1 Trauma and Orthopaedics, Hull University Teaching Hospitals NHS Trust, Hull, GBR; 2 Orthopaedics and Traumatology, Anassa Private Clinic, Volos, GRC

**Keywords:** soft tissue tension, intertrochanteric fracture, liner dissociation, dislocation, recurrent instability, total hip arthroplasty

## Abstract

Dislocation and instability of primary total hip arthroplasty (THA) is the leading cause for revision surgery, linked with significant financial burden and patient dissatisfaction. It is a multifactorial complication and requires accurate diagnosis and identification of the causative factors, as well as good preoperative planning for revision surgery. Despite all the best efforts, revision can lead to disappointing and frustrating results. We present a complex case of recurrent THA instability that required multiple operations before the identification of the main cause led to a satisfactory outcome. In addition, this is, to our knowledge, the first report of an Aesculap Plasmacup (Aesculap, Tuttlingen, Germany) liner dissociation and the first case where a previously well-united intertrochanteric fracture has been directly linked to recurrent instability. We aim to raise awareness of the complexity of such complications and the need for careful assessment of all the possible causes.

## Introduction

Dislocation of primary total hip arthroplasty (THA) is a fairly common complication [1], but recurrent instability has been mainly linked to revision surgery and salvage THA for femoral neck fractures and distorted anatomy especially due to dysplasia [2-4]. Polyethylene liner dissociation is a well-described complication of cementless acetabular cups, especially the Harris-Galante (Zimmer, Warsaw, Indiana) and the Pinnacle cups (DePuy, Warsaw, Indiana) [5-7]. However, it has been reported to occur late and has been attributed to wear and/or fracture of the locking mechanism [5-7]. We present a case of recurrent instability and dislocation, associated with polyethylene dissociation, that was quite challenging and required three reoperations and out-of-the-box thinking before it was resolved. To our knowledge, such a case has not been reported in the literature. Our aim is to draw attention to the need for careful preoperative planning of THA after a healed intertrochanteric fracture in order to prevent similar complications.

## Case presentation

**Figure 1:**
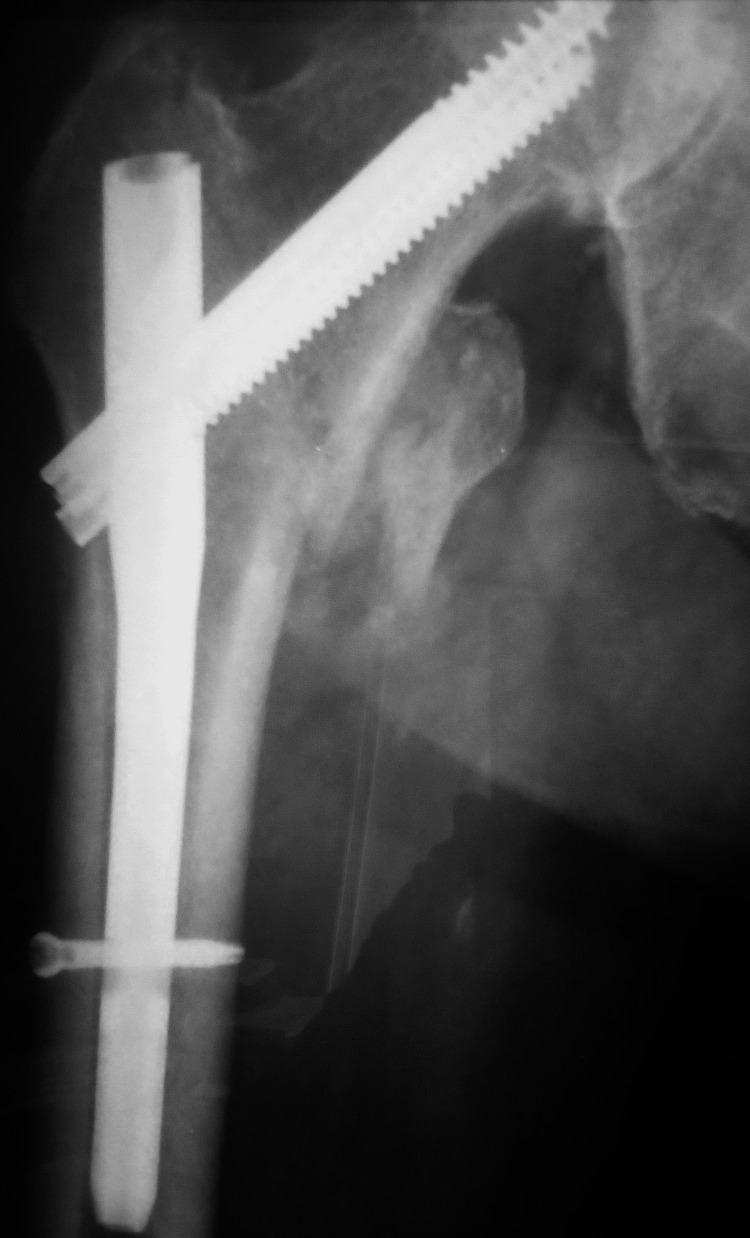
Healed intertrochanteric fracture three years before total hip arthroplasty

An 82-year-old man presented with a painful and clanking right hip two years post THA. Five years earlier, he sustained an intertrochanteric fracture that was successfully treated at a local hospital with a trochanteric nail (Figure 1).

**Figure 2:**
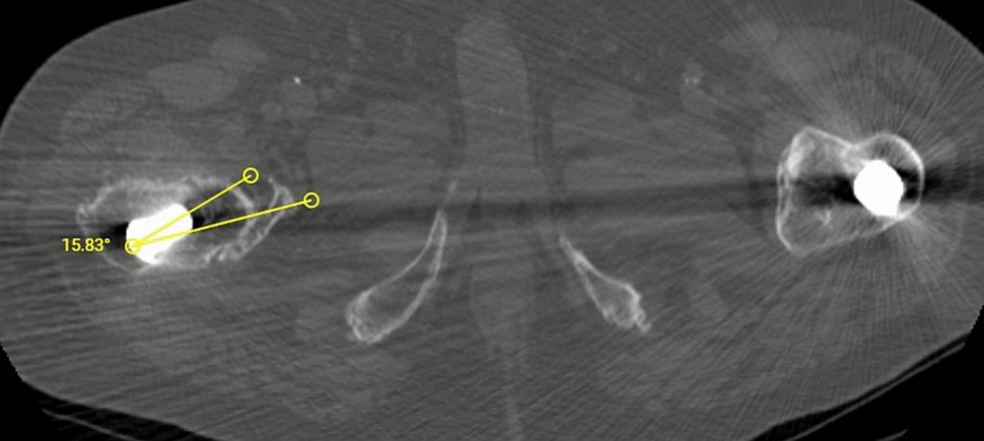
Extreme stem anteversion 15.8^o ^of anteversion

**Figure 3:**
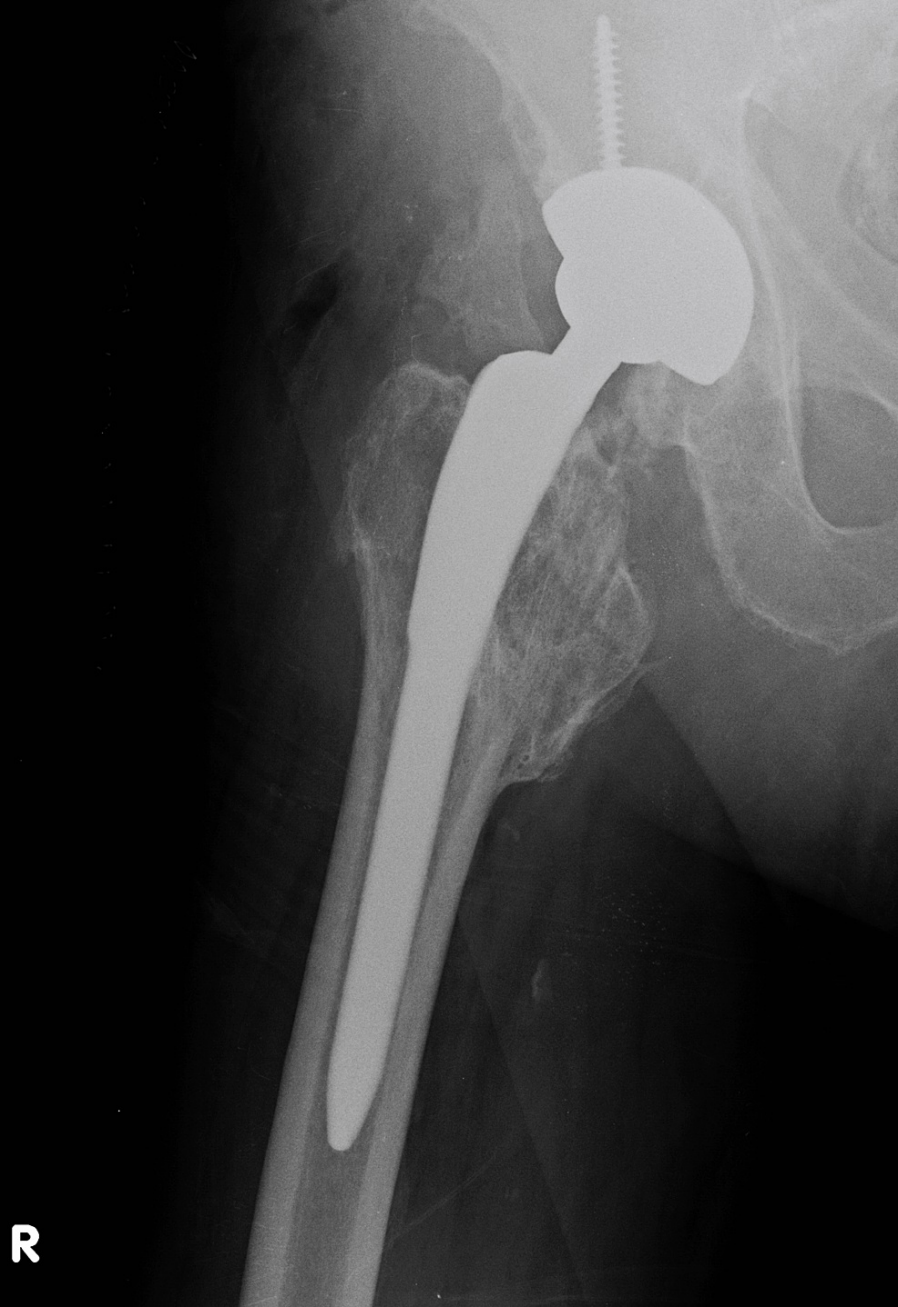
An obviously small offset partly due to anteversion

**Figure 4:**
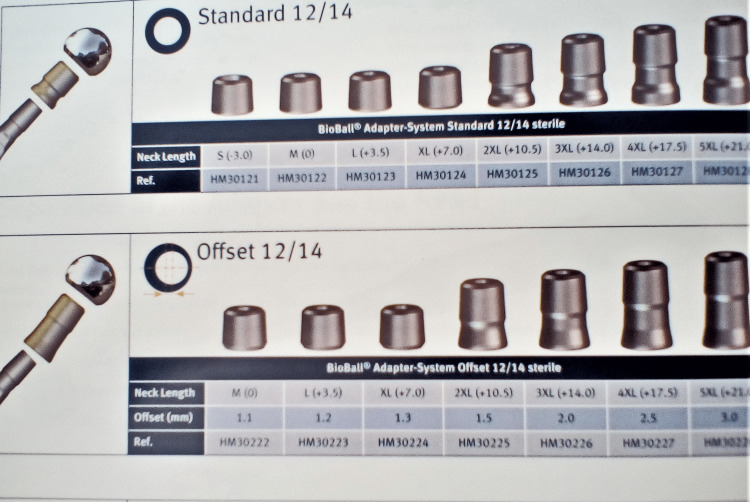
Merete BioBall offset neck inventory; the 2XL neck was used

**Figure 5:**
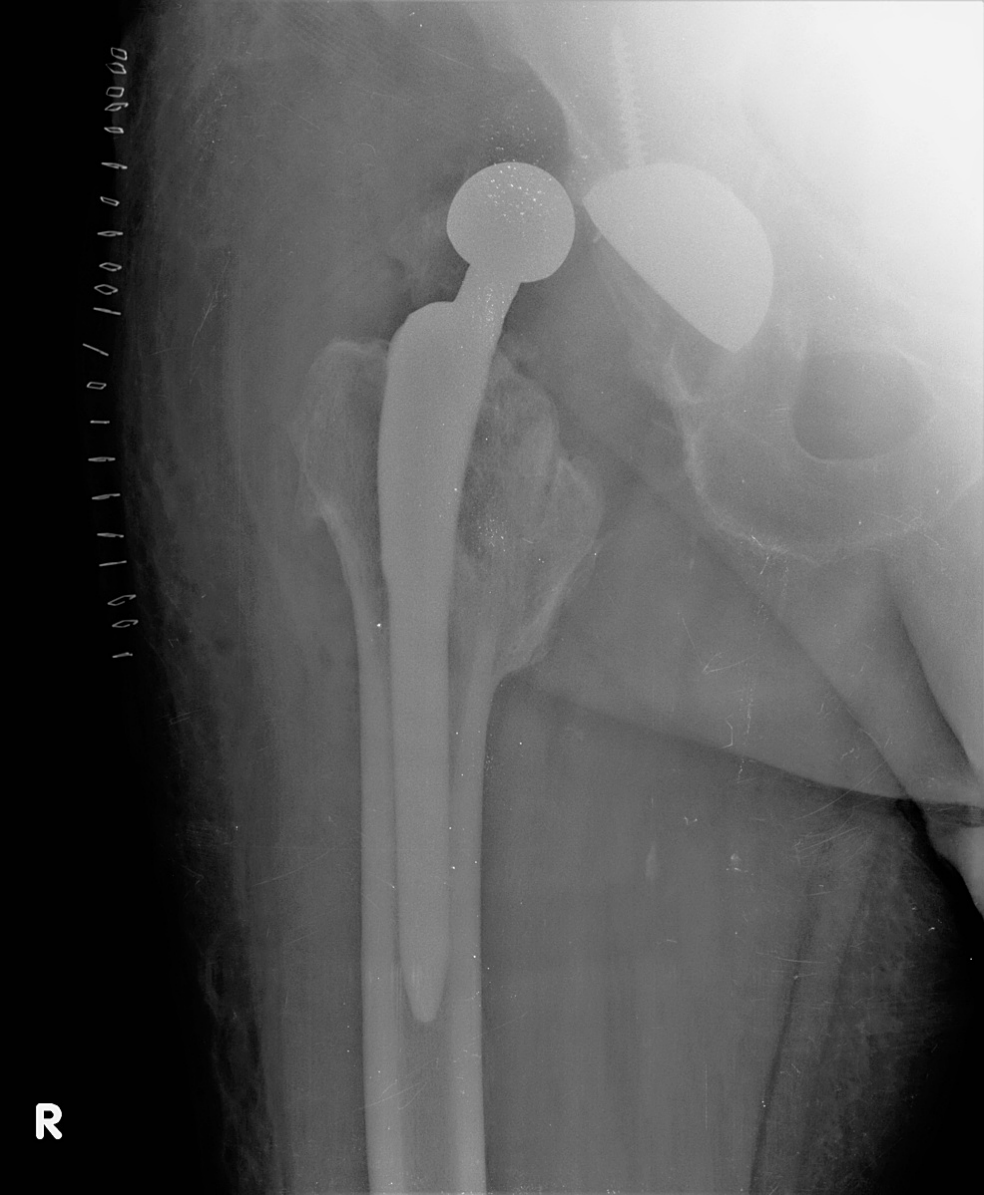
Immediate postoperative dislocation following first revision

Three years after the fracture, at the same hospital, he underwent cementless THA for osteoarthritis, which dislocated immediately postoperatively due to a reported fall, and was treated with closed reduction and six-week bed rest. His current symptoms started 18 months post THA. On examination, obvious anterior subluxation and a metallic noise were noted. Radiographic investigation, including a CT scan, revealed increased stem anteversion (15,8^o^) and a smaller offset partly due to the anteversion (Figures 2-3). Revision surgery was undertaken to increase offset and retroversion of the femoral head since the stem seemed adequately fixed. On operation, the polyethylene insert was surprisingly found dislodged from the metal Plasmacup SC (Aesculap, Tuttlingen, Germany), causing subluxation and noise. It was removed and a new liner was inserted firmly in the pristine looking metal cup with the extended rim positioned superoanteriorly. The femoral head (32mm, +7mm) was replaced with a new one (32mm, +0) with the interposition of an offset (+10.5mm) BioBall (Merete, Berlin, Germany) neck giving as much retroversion and varisation as possible (actual offset increased approximately 4,5mm) (Figure 4). Following a difficult reduction, the hip felt stable through a full range of movements. Unfortunately, on the third postoperative day, the hip dislocated anteriorly while the patient tried to turn in bed (Figure 5). There was a strong suspicion of a new liner dislodgement due to a clanking noise during reduction.

**Figure 6:**
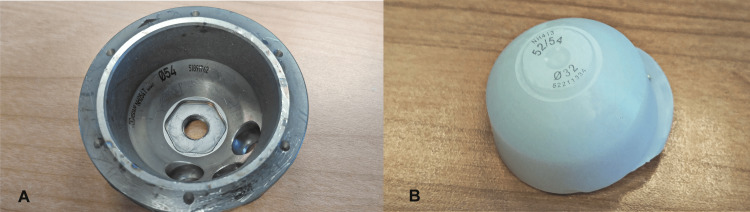
Removed metal shell (A) and second polyethylene liner (B) showing no signs of wear of the taper-lock locking surfaces

**Figure 7:**
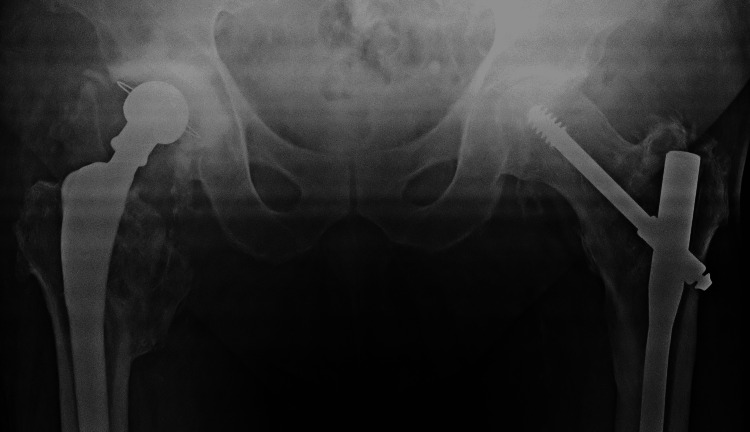
Second revision with a cemented cup and the 3XL neck (+14,0mm)

On re-operation, the polyethylene was indeed dislodged and a change of the acetabular implant to a cemented one was decided, although it showed no wear (Figure 6). The new cup was placed in a neutral position regarding anteversion and more closed superiorly. An even longer, offset neck (+14mm) was used, again in a retroverted and varus position (Figure 7). The reduction proved difficult and the hip was stable despite forceful rotation and even levering.

**Figure 8:**
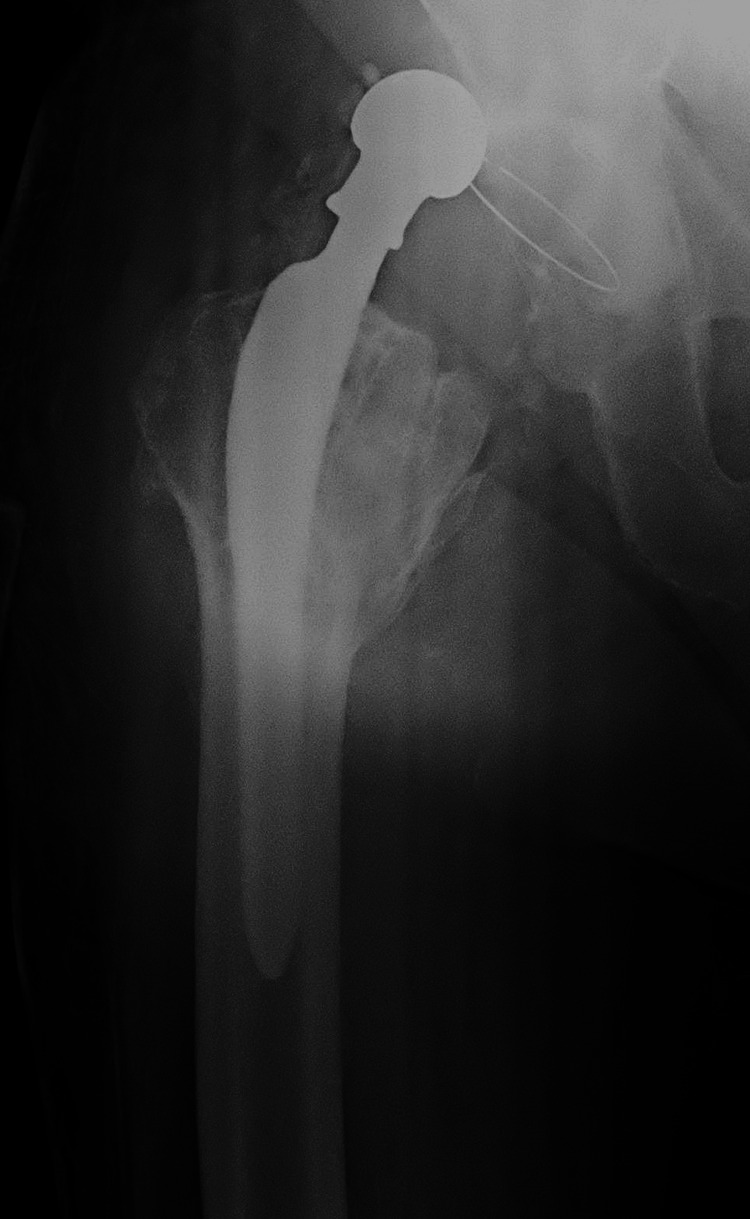
New dislocation five weeks following second revision

**Figure 9:**
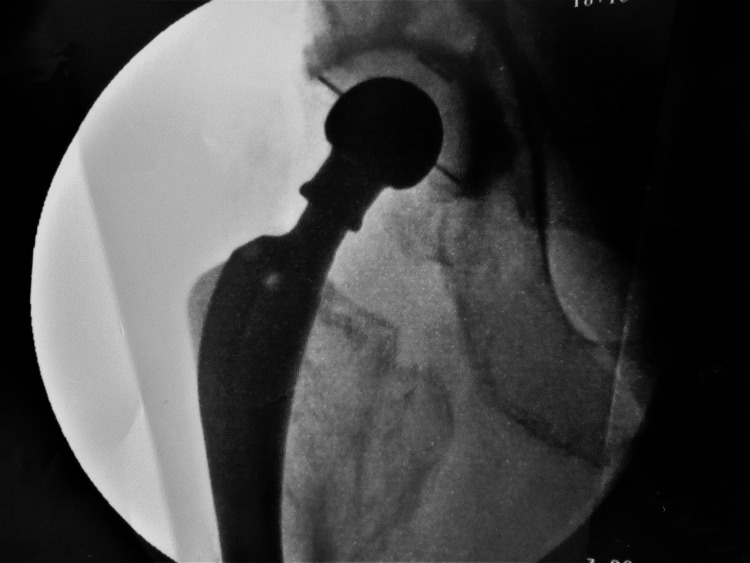
Successful closed reduction

**Figure 10:**
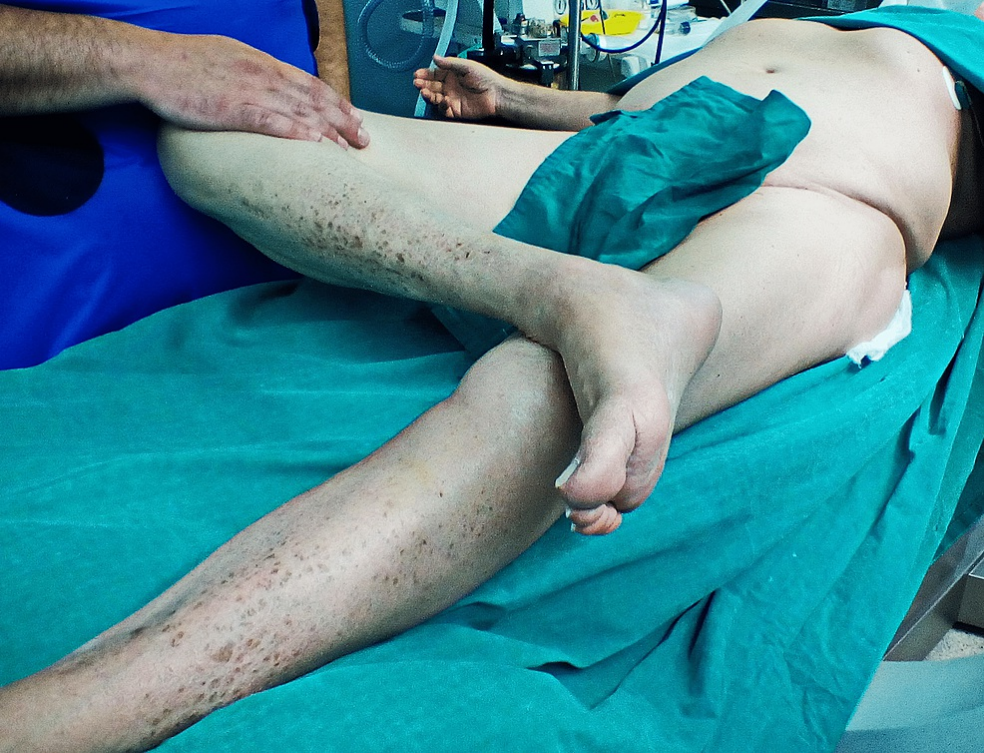
Unsuccessful attempt to re-dislocate in a figure-of-four position

Following four weeks of rehabilitation, the patient was able to walk and manage stairs without discomfort or signs of instability. At five weeks, he suffered an anterior subluxation in bed at home, that was reduced with gentle internal rotation of the leg. He was taken to the operating theatre, where under fluoroscopy, the hip was taken through a full range of movements, with levering in a figure-of-four position, but it proved impossible to dislocate. The following day, while attempting to stand up from a sitting position, he sustained an anterior dislocation (Figure 8). This was reduced under sedation and a new attempt to dislocate the hip even in a forced figure-of-four position proved unsuccessful (Figures 9-10). After careful evaluation, with a new CT scan, of the altered hip mechanics due to the previous fracture, the iliopsoas tendon was deemed to be the culprit and it was severed through a small incision. At five years of follow-up, no more dislocations have occurred.

## Discussion

THA Instability and dislocation is the first indication for revision surgery, with a frequency that is on the rise over the past decade [8]. It is associated with a great impact on quality of life, mortality, and treatment costs [9]. Being a multifactorial problem, with patient, surgeon, and implant parameters involved, it presents significant treatment challenges [2-4]. Cementless acetabular cups with a polyethylene insert have added a new instability cause, insert dissociation, due to locking mechanism failure [5-7]. Our patient combined several predisposing factors for instability but treating the most obvious ones did not resolve the problem. We had to reconsider every aspect in order to identify the main causative factor.

Regarding the patient himself, advanced age was the only negative parameter for instability. Although THA following hip fracture is associated with high dislocation rates, all studies on the subject refer to salvage THA due to internal fixation or hemiarthroplasty failure [10-13]. Our patient had a previous intertrochanteric fracture that was successfully treated and united and underwent THA for osteoarthritis three years later. The approach had been direct lateral; a well-positioned acetabular cup and a 32mm femoral head were both factors preventing instability. Wallner et al. [14] have identified that the combination of two or more of the following three radiological poor positioning factors predisposes to instability following THA: A cup placed at <35o or >55o, leg-length discrepancy >10mm, and difference in offset >10mm. Our patient had significantly lower offset, no leg-length discrepancy, and no cup malpositioning. Instead, he had 15,8^o^ of stem anteversion, an instability predisposing factor identified by Daly and Morrey [15], who suggested that it becomes significant only in combination with other factors.

**Figure 11:**
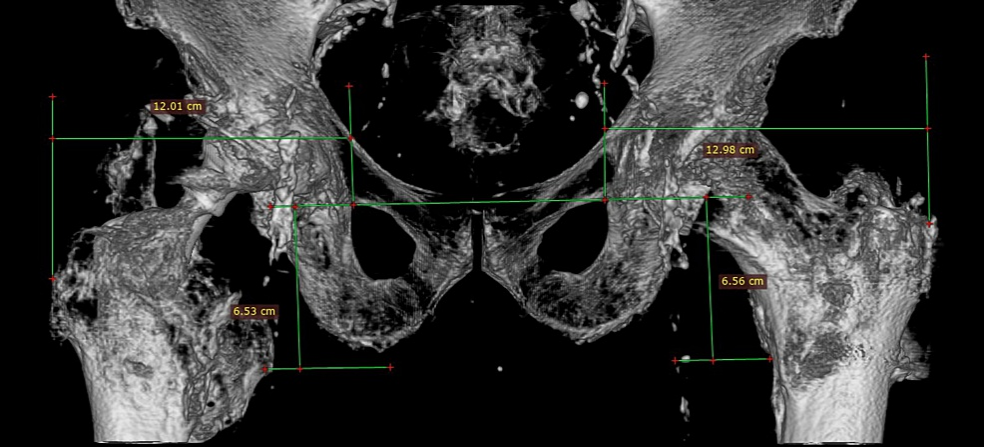
Offset and length measurements Offset restored to <10mm. Length, or vertical offset, measured in reference to the lesser trochanter, is very close on both sides, but on the THA side, we added 10mm since this was the calculated initial migration of the lesser trochanter following the trochanteric fracture (see Table 1).

**Figure 12:**
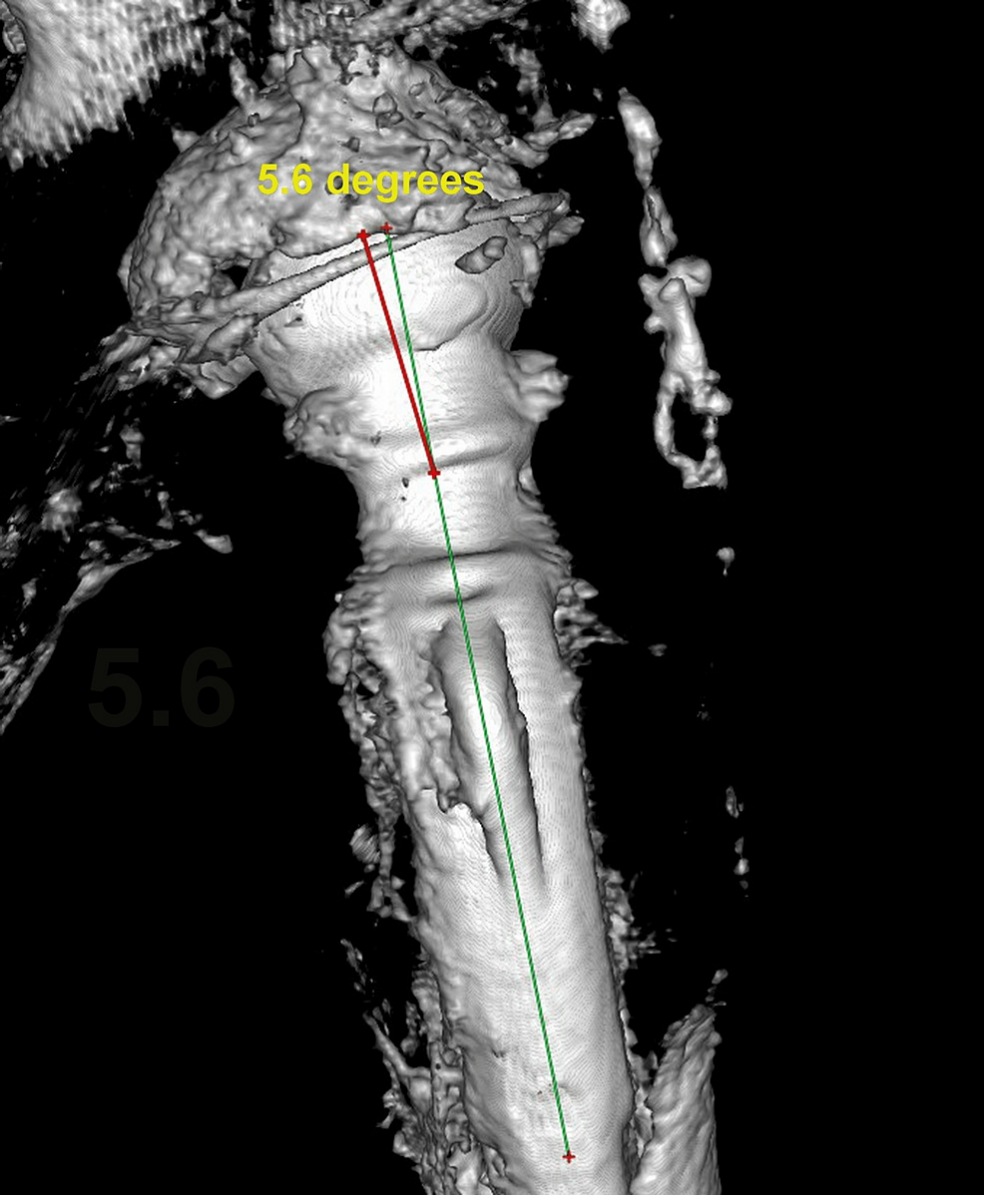
3D reconstruction with direct lateral view of the stem and neck showing that the Merete neck corrected anteversion by 5.6 degrees

**Figure 13:**
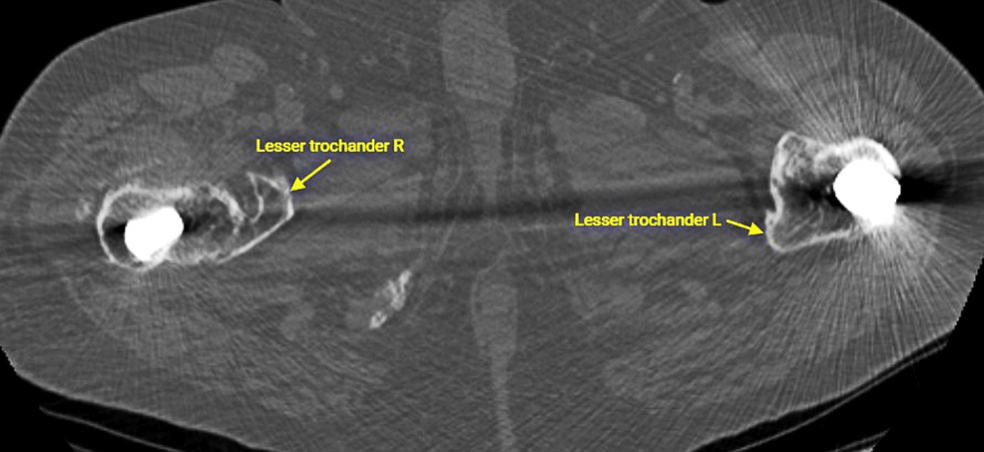
Extreme anterior position of the lesser trochanter due to the previous fracture. On the contralateral side, the intertrochanteric fracture did not involve the lesser trochanter that lies in its usual location

Our initial assessment was to increase the offset and reduce the stem anteversion that we considered responsible for the instability. Since the stem was well fixed, we elected to use an offset Merete BioBall neck that allows for adding varus and retroversion to an existing stem. Intraoperatively, however, we faced the unexpected problem of liner dissociation and with a pristine-looking metal shell, we opted for liner exchange. Liner dissociation has been reported with certain acetabular implants and is always related to the failure of the locking mechanism [5-7]. There has never been a report of this complication with the Aesculap Plasmacup; no locking mechanism problem was seen intraoperatively and the new liner locked firmly in place without any motion detected. Despite increasing the offset by approximately 4,5mm, reducing the anteversion, and with a new stable liner in place, a dislocation occurred in bed just three days postoperatively. A strong suspicion of repeated liner dissociation led to re-revision that confirmed the dissociation and, despite having a pristine looking cup, we elected to replace it with a cemented one in order to avoid re-dissociation. Additionally, we increased the offset to a degree that trial reduction was extremely difficult and dislocation for final head and neck insertion was impossible without a hook pulling anteriorly. However, despite a successful rehabilitation, five weeks down the line, a subluxation and an anterior dislocation occurred. Following reduction and being unable to manually dislocate the hip under anesthesia, despite forceful levering, we engaged in a new thinking process, with a new CT scan, trying to understand the biomechanics of this hip. Measurements, as seen in Table 1, showed that the offset had been restored to <10mm from the contralateral hip, and anteversion reduced to approximately 10^o^ (Figures 11-12). There was no clinical leg length discrepancy of more than 5mm following the initial THA and more than 10mm following the final revision. The only difference with the other hip was the position of the lesser trochanter that healed anteriorly due to the previous intertrochanteric fracture (Figure 13).

**Table 1:** Comparison of measurements between initial total hip arthroplasty (THA) and final revision THA with the contralateral, normal hip * We added 10mm on the THA side because the calculated proximal lesser trochanter migration was approximately 10mm

	RIGHT HIP	LEFT HIP - NORMAL
Acetabular anteversion Initial/Final	9.5^o^/3^o^	19^o^
Neck - Stem anteversion Initial/Final	15.8^o^/10.2^o^	9.8^o^
Combined anteversion Initial/Final	25.3^o^/13^o^	28.8^o^
Femoral offset Initial/Final	11.3mm/12.1mm	12.98mm
Leg length Initial/Final	Clinical discrepancy ~10 mm 7.30mm/7.53mm^*^	6.56mm

**Figure 14:**
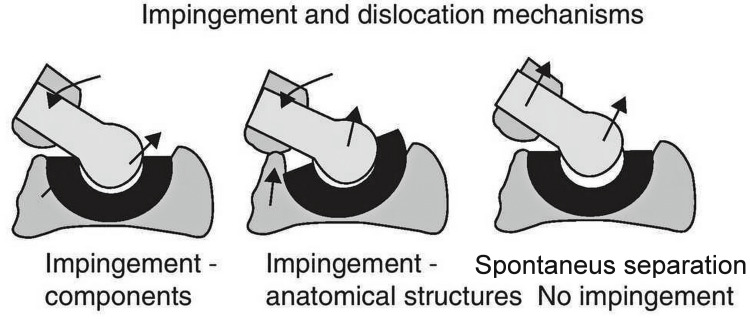
The three modes of dislocation Described by Scifert et al. [16], the first two are caused by impingement (no impingement was seen in our patient). The third one is spontaneous separation (as in our case) but Fernández-Fairen et al. [17] identified no spontaneous dislocation with a 32mm head. Image courtesy: Fernández-Fairen et al. [17]

We took into account the fact that, intra-operatively, we could only dislocate the hip by pulling anteriorly with a hook and that the patient suffered anterior dislocation trying to stand from a sitting position. Therefore, we formulated the hypothesis that, following fracture healing, the lesser trochanter healed in an anterior and proximal position. Over the next three years, the iliopsoas had shortened and exerted their pull to the femur abnormally. During THA surgery, the lesser trochanter position must have led the surgeon to place the stem in significant anteversion. Due to this anteversion and the abnormal forces exerted by the iliopsoas, the liner became loose and led to the symptoms of instability. It is also questionable whether the reported fall that led to the first dislocation was the cause of it, or could it have been the result of instability. Trying to fix the offset and the anteversion, we inadvertently increased the iliopsoas tension and, although passive dislocation was not possible, the tendon, under certain conditions (flexion-external rotation) could actively pull the head anteriorly and out of the cup, as in the spontaneous separation mode of dislocation as described by Scifert et al. [16]. However, Fernández-Fairen et al. [17] identified no spontaneous dislocation with a 32mm head but only with impingement, predominantly bony (Figure 14). We believe that even if no dislocation occurred, the concentration of those forces could eventually lead to cup wear and/or loosening in a few years. Taking this into account, we released the iliopsoas through a small incision. It felt bone-hard on palpation and made a snapping sound when released. This solved the problem since our patient had no episodes of subluxation, dislocation, or any instability during the five-year follow up.

## Conclusions

This is a complex case of THA recurrent instability and dislocation. To our knowledge, It is also the first report of liner dissociation of an Aesculap Plasmacup, that was the result of increased iliopsoas tension and not the primary cause of instability. Moreover, it points out that not only failed intertrochanteric fracture osteosynthesis, but also the successful treatment of such fractures, can lead to anatomical changes at the hip, influencing implant positioning as well as soft tissue laxity or tension, and ultimately leading to instability. Taking that into account, the altered hip anatomy during primary surgery could prevent future complications. Finally, dealing with THA recurrent instability requires extensive analysis of all relevant aspects for a successful treatment outcome.
